# A Rare Mechanism of Inhalation Injury: Direct Thermal Damage to the Lower Airway

**DOI:** 10.7759/cureus.44524

**Published:** 2023-09-01

**Authors:** Bayli P Davis, Alan Pang, Robyn Tapp, Catherine Anding, John Griswold

**Affiliations:** 1 Pulmonary and Critical Care Medicine, Texas Tech University Health Sciences Center, Lubbock, USA; 2 Surgery, Texas Tech University Health Sciences Center, Lubbock, USA

**Keywords:** burns airway, prognostic indicator, abbreviated injury score, thermal injury, modified baux score, bronchoscopy, inhalation injury

## Abstract

In patients with inhalation injury associated with major burns, the primary mechanism of tissue harm depends on the location within the respiratory tract. Proximal to the trachea, the upper respiratory tract epithelium is classically injured via direct thermal injury. Such injury occurs due to the inhalation of high-temperature air. These upper airway structures and the tracheobronchial tree’s dense vasculature protect the lower airways and lung parenchyma from direct thermal damage. The lower respiratory tract epithelium and lung parenchyma typically become injured secondary to the cytotoxic effects of chemical irritants inhaled in smoke as well as delayed inflammatory host responses. This paper documents a rare case in which a patient demonstrated evidence of direct thermal injury to the lower respiratory tract epithelium. A 26-year-old Caucasian male presented to the emergency room with 66% total body surface area thermal burns and grade 4 inhalation injury after a kitchen fire. Instead of visualizing carbonaceous deposits in the bronchi, a finding common in inhalation injury, initial bronchoscopy revealed bronchial mucosa carpeted with hundreds of bullae. Despite the maximum grade of inhalation injury per the abbreviated injury score and a 100% chance of mortality predicted with the revised Baux score, as well as a clinical course complicated by pneumonia development, bacteremia, and polymicrobial external wound infection, this patient survived. This dissonance between his expected and observed clinical outcome suggests that the applicability of current inhalation injury classification systems depends on the precise mechanism of injury to the respiratory tract. The flaws of these grading scales and prognostic indicators may be rooted in their failure to account for other pathophysiologic processes involved in inhalation injury. It may be necessary to develop new grading and prognostic systems for inhalation injury that acknowledge and better account for unusual pathophysiologic mechanisms of tissue damage.

## Introduction

Inhalation injury is defined as damage to the respiratory system secondary to thermal or chemical insults encountered during inspiration [1, 2]. In inhalation injuries secondary to fire with prolonged smoke exposure, the respiratory system can become compromised because of insults to the upper respiratory tract (anatomical structures proximal to the trachea) as well as the lower respiratory tract (trachea, bronchi, bronchioles, alveolar duct, and alveoli) [3]. Historically, direct thermal injury only occurs in the upper airway due to the efficient warming and cooling of inhaled air by the tracheobronchial circulation. Most gases have reached body temperature once distal to the glottis. In contrast, the lower respiratory tract and more distal pulmonary structures, such as the trachea, bronchi, bronchioles, and lung parenchyma (alveoli), are typically injured from a cytotoxic cascade of events secondary to gaseous particles inhaled in smoke. Toxic gases or fumes are formed from the decomposition of matter during the process of combustion [2, 4]. In these subglottic regions of the respiratory tract, smoke inhalation causes chemical irritation, culminating in the release of neuropeptides from the richly innervated tracheobronchial tissues. These neuropeptides ignite an inflammatory cascade of bronchoconstriction, increased vascular permeability, formation of reactive oxygen species (ROS), and vasodilation. These factors lead to local hyperemia, which facilitates the delivery of polymorphonuclear leukocytes and cytokines to the injured tissue. ROS and disruption of the bronchial epithelium lead to trans-vascular migration of plasma proteins and fluid into the alveoli and bronchial mucosa. The resulting mucosal exudate and associated pulmonary edema cause decreased pulmonary compliance, inactivation of surfactant, and regional atelectasis. All these factors contribute to a ventilation-perfusion mismatch with varying degrees of hypoxemia and worsening pulmonary functionality [2, 4].

Fiberoptic bronchoscopy remains the gold standard for confirmatory diagnosis of inhalation injury. Classic findings include varying degrees of erythema, edema, mucosal ulceration and erosions, hemorrhages, bronchial secretions, soot deposits, and tissue necrosis, with different grades of severity showing distinct macroscopic changes on bronchoscopic evaluation [5, 6]. The abbreviated injury score (AIS) is a widely accepted method of severity assessment and is based merely on such bronchoscopy findings. According to this grading scale, inhalation injuries can range from 0 (no injury; characterized by the absence of carbonaceous deposits, erythema, edema, bronchorrhea, or obstruction) to grade 4 (massive injury; characterized by mucosal sloughing, necrosis, and endoluminal obstruction) [5, 7]. Despite diagnostic utility, the severity of inhalation injury based on the AIS is an imperfect predictor of patient mortality [8]. The most reliable prognostic indicator used currently is the revised Baux score, which calculates a burn patient’s mortality as R-Baux score = (TBSA + age + [17×R]), where R = 1 or 0 if inhalation injury is present or not present, respectively [9].

After securing the airway, management of inhalation injury is primarily centered on supportive measures that target bronchospasm, pulmonary secretions, and foreign materials causing airway obstruction, and atelectasis [5]. If wheezing or bronchospasm is present, aerosolized bronchodilators are indicated as they decrease airway resistance and help clear mucus [10]. Mucolytic agents help prevent obstruction of the airway by clearing out fibrin casts and sloughed epithelium. These agents include aerosolized acetylcysteine, which should be used with caution due to the potential for bronchoconstriction. Aerosolized heparin can also be used as it inhibits fibrin cast development [11, 12]. Chest physiotherapy and early ambulation are crucial adjuncts to treatment, while prophylactic antimicrobial therapy, corticosteroids, and early tracheostomy placement are strategies that have failed to demonstrate a consistently significant impact on survival. Clear outlines of the parameters as well as mode of mechanical ventilation in the context of inhalation injury are not well established. Lower tidal volumes and plateau pressures as much as tolerated by the patient are the general recommendation, although a large retrospective study from 2015 suggested that higher tidal volumes decreased the risk of acute respiratory distress syndrome (ARDS), risk of atelectasis, and number of ventilator days in pediatric patients with inhalation injury [13].

This paper highlights a unique case of severe inhalation injury in which a patient demonstrated bronchoscopic evidence of direct thermal damage to the respiratory tract epithelium distal to the larynx. We contrast his expected prognosis with his observed clinical outcome, aiming to elucidate areas of weakness in the current diagnostic and prognostic methods used in inhalation injury.

## Case presentation

**Figure 1:**
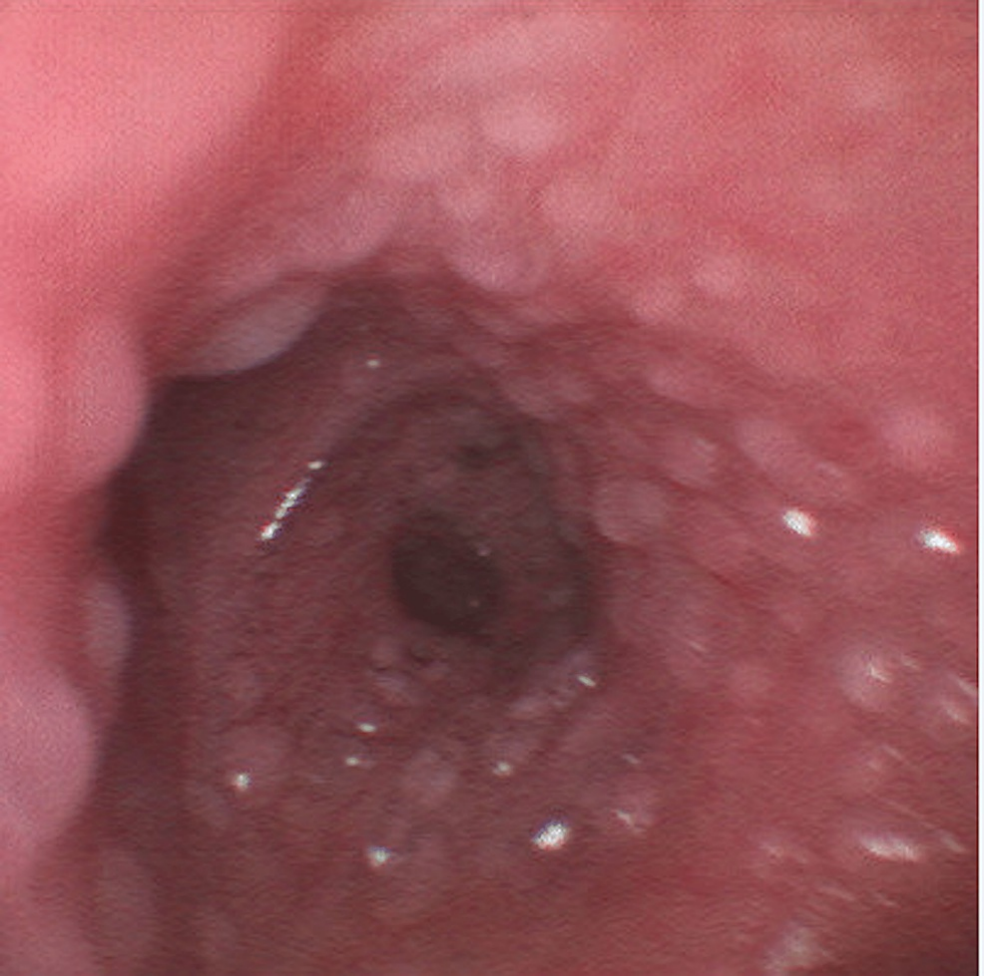
Unruptured bullae carpeting the tracheobronchial epithelium.

**Figure 2:**
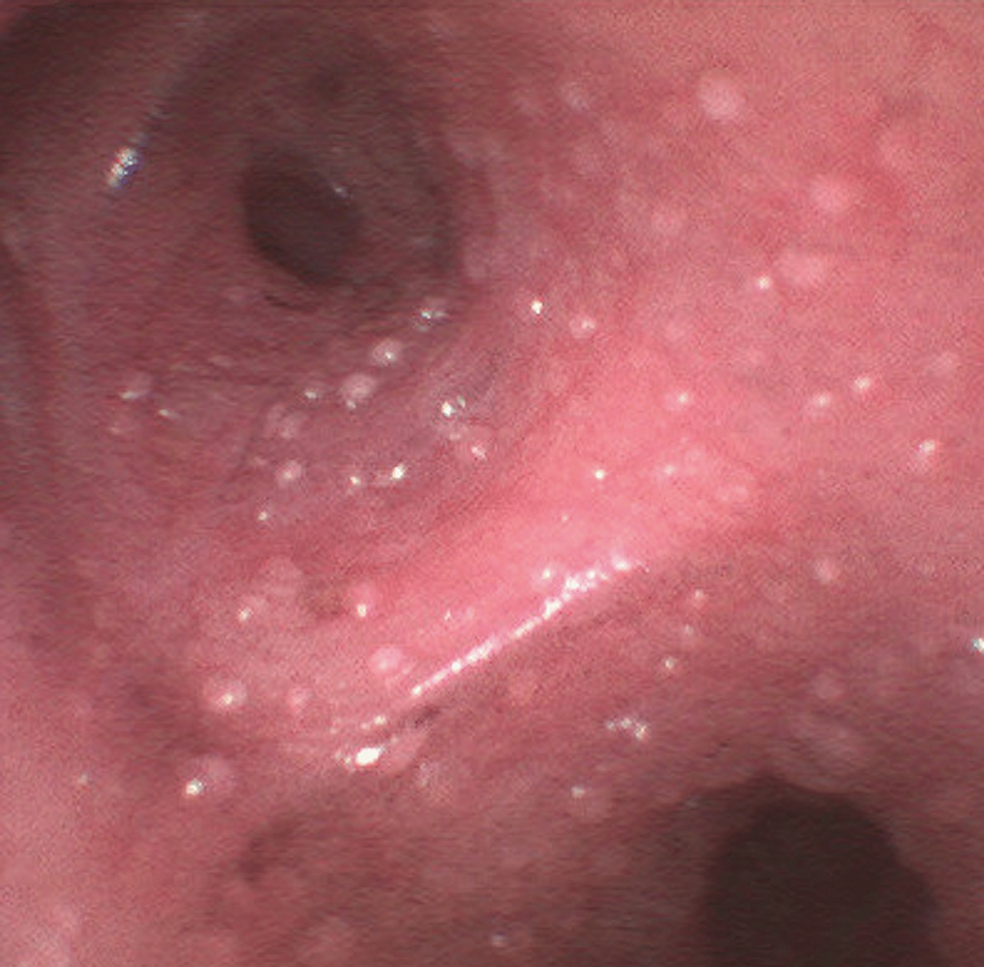
Unruptured bullae carpeting the tracheobronchial epithelium.

**Figure 3:**
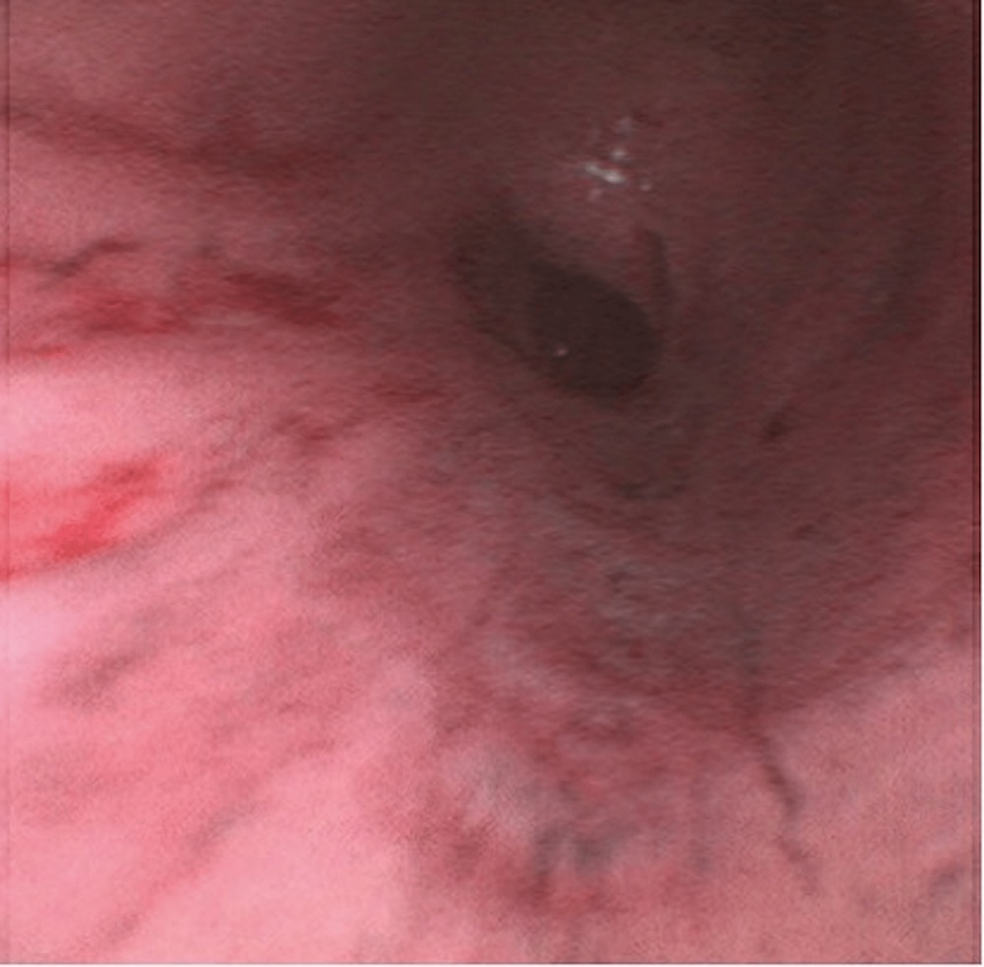
The blistered mucosa present on the day of admission had sloughed off, leaving behind denuded, erythematous distal airway epithelium.

A 26-year-old Caucasian male presented to the emergency room with 66% total body surface area (TBSA) second and third degree thermal burns and grade 4 inhalation injury after an accident at home. The patient's recollection of the incident was limited, but the fire apparently started after he lit a candle. The patient was found by emergency medical personnel with altered sensorium, alight within the structure. He was intubated upon arrival to the emergency department. A primary survey in the trauma bay revealed an intact airway, bilateral breath sounds, normothermia, and adequate perfusion. There were burns to the face, bilateral upper extremities, bilateral lower extremities, and back. Lungs were clear to auscultation bilaterally, respirations were non-labored, and the chest exhibited symmetric expansion. The patient was given 16 L lactated ringers, 1.2 L of 5% albumin, and 5 L of plasma over the first 24 hours in line with the Parkland formula; subsequent fluid replacement was adjusted in response to his urine output. He was treated with our hospital’s inhalation injury protocol including aerosolized heparin and acetylcysteine as well as weaning of ventilatory support as tolerated. Initial bronchoscopy on the day of admission revealed the findings shown in Figures 1, 2. A bronchoscopy performed on hospital day two revealed the findings shown in Figure 3.

The patient underwent emergent escharotomies of the bilateral upper extremities within the first 24 hours of admission. Subsequent surgical intervention was postponed until after extubation. The patient’s extensive injuries paired with his altered sensorium made it difficult to liberate him from the ventilator. As such, he required mechanical ventilation for seven days. His burn care then proceeded with excision and wound coverage of external burns. This included the application of a biodegradable temporizing matrix as well as autologous skin cell suspension (ReCell), and subsequent split-thickness skin grafting when appropriately integrated over the course of several operations. The patient’s hospital course was complicated by ventilator-acquired pneumonia, bacteremia, and external wound infection with respiratory, blood, and wound cultures growing *Klebsiella pneumonia/Streptococcus pyogenes*, *Klebsiella pneumonia/Enterobacter cloacae*, and *Enterobacter cloacae/Staphylococcus aureus/Pseudomonas putida*, respectively. He was treated with intravenous (IV) cefepime for five days followed by two days of azithromycin once sensitivities resulted. In addition to numerous surgeries and intravenous antibiotics, his external wounds were treated with local wound care and standard antimicrobial dressings. After a prolonged hospital stay of 73 days as well as aggressive physical and occupational therapy, he was discharged to a local skilled nursing facility with 3% total body surface area (TBSA) burns.

## Discussion

It is important to consider the specific context of our patient’s inhalation injury, given that the degree of tissue damage as well as the precise pathophysiology of inhalation injury is highly multifactorial [5]. Unfortunately, the patient’s altered mentation at the time of initial medical contact, as well as his inability to recall the events in the period immediately surrounding the accident preclude certain details of our patient’s case, such as the duration of fire and smoke exposure as well as the precise materials of combustion. Since the fire occurred within the enclosed space of the patient’s home, it is likely that the main components of inhaled gas were combustion products of the burning house furniture. However, the absence of soot deposits noted on the initial bronchoscopy implies that the materials of combustion were somewhat poor in terms of carbon and that particulate matter comprised an insignificant percentage of matter inhaled during the incident. The chronology of bronchoscopy findings provides a further clue about the mechanism behind the inhalation injury in our patient. It has been previously established that when inhalation injury occurs via the traditional mechanism of chemical irritation and cytotoxicity, bronchoscopy will show denuding of the respiratory mucosa and sloughing of airway tissue. In our case, the initial bronchoscopy from the day of admission showed bullae lining the epithelial mucosa. Denuded tissue and evidence of mucosal sloughing were not seen until subsequent bronchoscopy on hospital day two. The appearance of bullae prior to any evidence of mucosal defoliation strongly suggests the presence of a separate, more acute, pathophysiologic mechanism of tissue injury in our patient.

The precise pattern of tissue changes that we observed within several hours of admission sheds light on the mechanism of distal airway injury involved in our patient's case. According to the modern-day standard classification of burn injuries, the presence of blistering in itself is a sufficient criterion for second-degree thermal burn [14]. Considering this definition, the carpet of bullae lining our patient’s distal airway epithelium is strongly suggestive of direct thermal injury. In 1945, Moritz et al. performed an experiment on dogs that explored the effects of inhaled heat on the airways and set the widely accepted precedent that lower airway damage can only be thermal in nature if this distal mucosa comes into direct contact with flame [15]. Nearly eight decades later, these authors’ claims have largely held true. With the exception of blast injuries or severe cases of steam inhalation - which our case showed no evidence of given the paramedics’ report of the scene as well as the patient’s history involving a lit candle - direct thermal injury to the lower airways has never been previously documented in cases of inhalation injury associated with major burns [2].

Taken with the current classification system of burn injuries, our observations refute the conclusions made years ago by Moritz, Henriques, and McLean. Our patient had evidence of second-degree burns to the distal airways within 24 hours of being found inside a burning house, which caught fire after a candle was lit. However, it is unclear as to exactly how enough heat was generated during this incident to cause this type of injury. Nonetheless, it appears that the temperature of inhaled smoke can in fact overcome the airway’s ability to diffuse heat adequately such that the tracheal and bronchial mucosa are injured thermally, even in the absence of direct contact with flame, steam, or explosive blasts. When this mechanism of injury happens, it occurs in addition to other pathophysiologic processes that have long been established. The existence of a novel mechanism of distal airway damage, as well as the specific way in which our patient’s inhalation injury manifested, gives rise to several important implications. Direct thermal damage may potentiate the effects of chemical irritation on the respiratory tissue and exacerbate delayed inflammatory responses evoked within the host following smoke inhalation. This heightened degree of tissue damage may lead to worsened clinical outcomes for victims of such injuries.

Given the potential for worsened prognoses, it is imperative to consider how unusual and unrecognized mechanisms of lower airway damage may challenge the current systems used to grade severity and predict mortality in this patient population. According to the AIS, our patient’s presentation aligned with a grade 4 (i.e. maximum grade) inhalation injury, which is traditionally characterized by necrotic mucosal epithelium and not a carpet of tense bullae, as in our case. Furthermore, the revised Baux score predicted his chance of mortality at 100%. While this grade of inhalation injury and poor estimated prognosis per the revised Baux score is not unique, the precise mechanism of injury to the proximal lower airways paired with the incongruence between the patient’s expected and observed outcomes is noteworthy. Importantly, our team’s management of this patient’s inhalation injury complied with the current standard of care [10-12]. The mortality implications of both inhalation injury and the pathologic sequela that arise secondary to it have long been established. A study published in 1987 found that while inhalation injury increases a burn patient’s mortality by up to 20%, inhalation injury complicated by pneumonia, as seen in our patient’s case, can increase the risk of death by 60% [16]. While debate persists as to whether the grade of inhalation injury via the AIS consistently correlates with mortality, alternatives to this method of severity assessment are scarce [7, 17, 18]. In congruence with other published work questioning the association between bronchoscopy-assigned grade of injury and prognosis, our case demonstrates that the former does not necessarily correlate with the latter.

## Conclusions

Although in traditional cases of inhalation injury, most gases have cooled enough before reaching the glottis such that direct thermal injury distal to this point is not commonly observed, burn specialists should be aware that cytotoxicity and the subsequent inflammatory cascade of events that follow are not the only mechanisms by which the tracheobronchial tree may be injured. In patients with significant TBSA burning and concomitant inhalation injury, it is important to consider thermal injury to the distal airway. This rare mechanism of injury presents earlier than traditional mechanisms (in terms of bronchoscopy evidence of tissue damage). Direct thermal injury may potentiate the known pulmonary sequela that later arises in this population and necessitate prolonged mechanical ventilation.

The applicability of the AIS and revised Baux score in gauging inhalation injury severity and prognosis appears somewhat dependent on the precise mechanism of injury to subglottic structures. Given this, it remains unclear how the potentiation of deleterious effects from both mechanisms of injury compares with those of either mechanism of injury (direct thermal injury versus cytotoxicity) in isolation. Our findings underscore the importance of developing improved grading and prognostic scales for inhalation injuries that account for the precise mechanism of injury to the respiratory system in its entirety.
